# Interplay of Prenatal and Postnatal Risk Factors in the Behavioral and Histological Features of a “Two-Hit” Non-Genetic Mouse Model of Schizophrenia

**DOI:** 10.3390/ijms21228518

**Published:** 2020-11-12

**Authors:** Yi-Chun Chang, Wai-Yu Li, Lukas Jyuhn-Hsiarn Lee, Li-Jen Lee

**Affiliations:** 1 Graduate Institute of Anatomy and Cell Biology, College of Medicine, National Taiwan University, Taipei 10048, Taiwan; r99446016@ntu.edu.tw (Y.-C.C.); aryu0410@gmail.com (W.-Y.L.); 2 National Institute of Environmental Health Sciences, National Health Research Institutes, Miaoli 35053, Taiwan; lukaslee@gmail.com; 3 Institute of Brain and Mind Sciences, College of Medicine, National Taiwan University, Taipei 10048, Taiwan; 4 Neurobiology and Cognitive Science Center, National Taiwan University, Taipei 10617, Taiwan

**Keywords:** risk factors, schizophrenia, stress-coping, ventral tegmental area (VTA), mPFC, dendritic structure

## Abstract

Schizophrenia is a multifactorial developmental neuropsychiatric disorder. This study examined the interplay of maternal infection and postweaning social isolation, which are prenatal and postnatal risk factors, respectively. Pregnant mice received poly I:C or saline injection on gestation day 9 and the pups were weaned at postnatal day 28. After weaning, male offspring were randomly assigned into group-rearing and isolation-rearing groups. In their adulthood, we performed behavioral tests and characterized the histochemical features of their mesocorticolimbic structures. The sociability and anxiety levels were not affected by either manipulation, but synergistic effects of the two hits on stress-coping behavior was observed. Either of the single manipulations caused defects in sensorimotor gating, novel object recognition and spatial memory tests, but the combination of the two hits did not further exacerbate the disabilities. Prenatal infection increased the number of dopaminergic neurons in midbrain, whereas postweaning isolation decreased the GABAergic neurons in cortex. Single manipulation reduced the dendritic complexity and spine densities of neurons in the medial prefrontal cortex (mPFC) and dentate gyrus. Our results support the current perspective that disturbances in brain development during the prenatal or postnatal period influence the structure and function of the brain and together augment the susceptibility to mental disorders, such as schizophrenia.

## 1. Introduction

Schizophrenia is a multifactorial developmental neuropsychiatric disorder. In patients of schizophrenia, the symptoms usually emerge during adolescence to young adulthood. The etiology of such psychiatric disorders has therefore been attributed to disrupted brain development [1,2,3]. Depending on different functional aspects, the critical time period for vulnerable brain development could be the perinatal or adolescent era, or both [4]. The two-hit hypothesis of schizophrenia had been proposed to model the pathogenic processes. It suggests that the first hit attacks the developing nervous system during the embryonic or perinatal period, and the second hit occurs during adolescence, which collectively lead to the onset of clinical symptoms [5,6,7,8]. Numerous two-hit animal models for schizophrenia have been developed [9,10,11,12]. Frequently, a genetic risk factor is combined with an environmental adversity [13,14,15,16]. However, genetic manipulation might have profound impacts on both the development and maintenance of the nervous system [17]; the critical temporal window of the genetic factor in the pathogenesis of mental disorders may not be clearly defined. To evaluate the two-hit hypothesis of schizophrenia, temporally well-controlled manipulations of risk factors during prenatal or postnatal eras are desirable. Environmental risk factors such as prenatal infection, maternal stress, neonatal maternal separation, postweaning isolation [3,18] can be manipulated at or during a specific time period.

Accumulating evidence suggests that maternal infection during pregnancy is associated with neurodevelopmental psychiatric disorders [19,20,21]. Exposure to infectious agents increases maternal cytokine production and enters the fetal circulation, which might affect the neurodevelopment processes of the fetus [22,23]. The double-stranded RNA viral mimic poly-inosinic/cytidylic acid (poly I:C) is commonly used to model the consequences of maternal immune activation [24,25,26]. Deficits in novel object exploration [27,28] impairment of prepulse inhibition (PPI) of the acoustic startle reflex [29] and increased sensitivity to dopamine (DA)-releasing psychostimulants [30] are observed in offspring born to poly I:C-exposed mothers, which support the use of prenatal infection as an animal model for schizophrenia [31].

Early-life adversity, such as social isolation, affects brain development and adult behaviors that are associated with schizophrenic symptoms [32,33,34]. In animal models of social isolation, hyper-reactivity to a novel environment [35], impairment of PPI [36], deficits in novel object recognition [37] and impairments in spatial learning and memory in the Morris water maze [38] are noticed. Early social experiences also significantly influence the neurochemical and anatomical development of the DA and γ-Aminobutyric acid (GABA) systems in the prefrontal cortex (PFC) [39]. Dopaminergic neurons in the midbrain ventral tegmental area (VTA) project to the PFC and hippocampus which play important roles in emotional and cognitive function. Alterations of this system are associated with symptoms in schizophrenic patients [40,41,42].

In this study, we aimed to examine the interaction of the two risk factors given during two definite time windows. In this regard, maternal infection and postweaning social isolation, representing prenatal and postnatal risk factors, respectively, were chosen to establish a two-hit non-genetic model for schizophrenia. In the present study, a single shot of poly I:C was administered to pregnant mice on gestation day (GD) nine to simulate prenatal infection (first hit) [26], and the pups were subjected to a postweaning social isolation paradigm [33] that replicates adolescent life adversity (second hit). Behavioral performances, density of DA neurons in the VTA and neuronal structure in the PFC and hippocampus were examined in this model. The individual and synergistic effects of the two hits in the mesocorticolimbic system were evaluated.

## 2. Results

### 2.1. Behavioral Examinations

**Figure 1:**
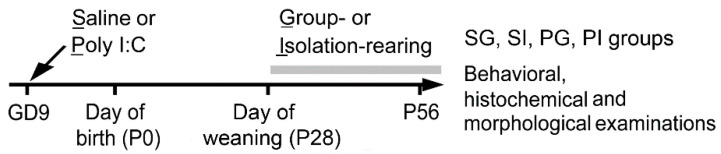
Experimental design. Saline or poly I:C (2 mg/kg) was intraperitoneally given to the pregnant mice at gestation day (GD) 9. On the day of weaning, postnatal day (PD) 28, male offspring were divided into groups or isolation-rearing groups. Four groups of animals were then examined after PD 56.

Male offspring born to saline- or poly I:C-treated mothers were raised in groups or individually (isolation) after the day of weaning, postnatal day (PD) 28. Four groups of animals (saline-group, SG; saline-isolation, SI; poly I:C-group, PG and poly I:C-isolation, PI) were reared and then subjected to behavioral, histochemical and morphological examinations (Figure 1).

**Figure 2:**
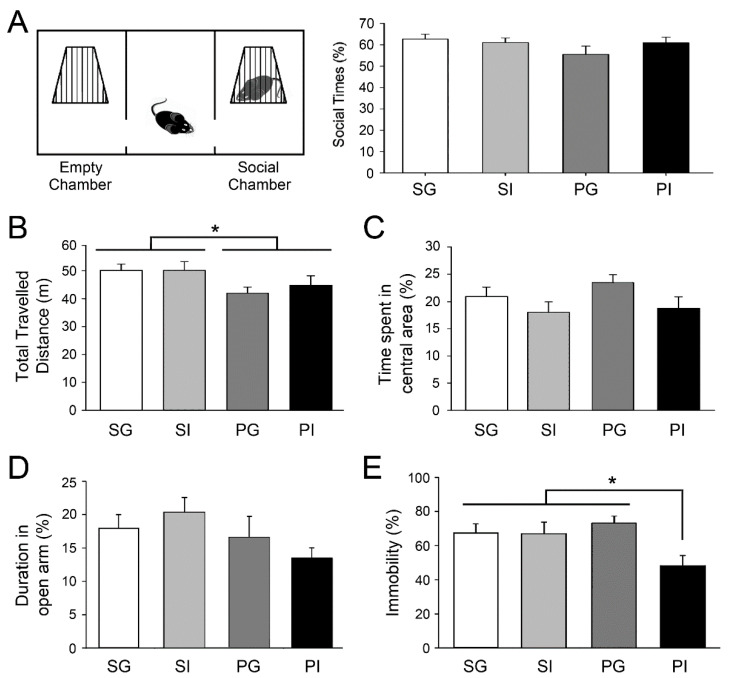
Examinations of social, locomotive and emotional behaviors. (**A**) Social behaviors were examined in a three-chamber test. (**B**,**C**) Locomotor activity of mice was examined in a novel open field. (**D**) The extent of anxiety was estimated in an elevated plus maze. (**E**) The degree of depression was evaluated by measuring the time spent in immobility in a forced swim test. Results are mean ± SEM (*n* = 8–12 per group). * *p* < 0.05.

Patients with schizophrenia display altered social, emotional and cognitive functions; we therefore first examined the behavioral performances of these aspects in young adult (~PD 56) mice. The social behaviors of mice were examined in a three-chamber test. All mice spent more time in the social chamber and the sociability was not affected by either manipulation (Figure 2A). We next examined the mice in an open field. Mice in the PG and PI groups exhibited reduced locomotor activity (Figure 2B) that was primarily attributed to maternal poly I:C exposure (F_(1,42)_ = 20.10, *p* < 0.001). Reduced locomotor activity may reflect anxiety or depression [43,44]. However, the duration of time spent in the central area of the open field did not significantly differ in all groups (Figure 2C), which indicated similar anxiety levels. This result was confirmed by the performances in an elevated plus maze. The time spent in the open arms, the index of anxiety, was comparable among all groups (Figure 2D). We also conducted a forced swim test, and the percentage of time spent in immobility was used as the index of depression [43] and stress-coping reaction [45]. Interestingly, mice in the PI group exhibited reduced immobility in this test (Figure 2E). A two-way ANOVA indicated that maternal poly I:C exposure and postweaning isolation produced this synergistic effect (df = 1, *p* = 0.027).

**Figure 3:**
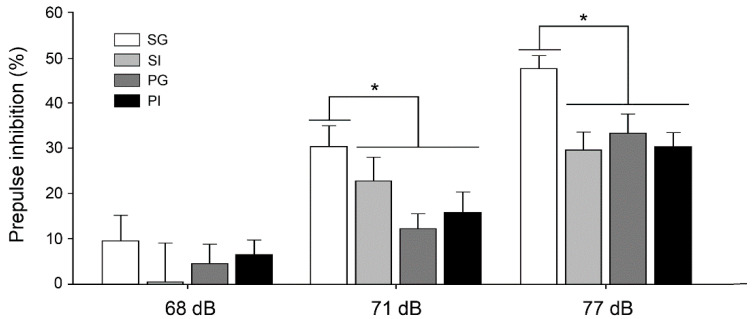
Behavioral examination of sensorimotor gating. The property of sensorimotor gating was assessed using prepulse inhibition (PPI) of the acoustic startle reflex. Results are mean ± SEM (*n* = 8–12 per group). * *p* < 0.05.

Patients with schizophrenia exhibit reduced sensorimotor gating ability which can be evaluated using the prepulse inhibition (PPI) test [46,47]. Significant reductions of PPI were observed in the SI, PG and PI groups compared to the SG group (Figure 3), which indicated that both manipulations affected the sensorimotor gating function in adult offspring (F_(2,39)_ = 74.47, *p* < 0.001). However, these effects were not aggravated in the two-hit model.

**Figure 4:**
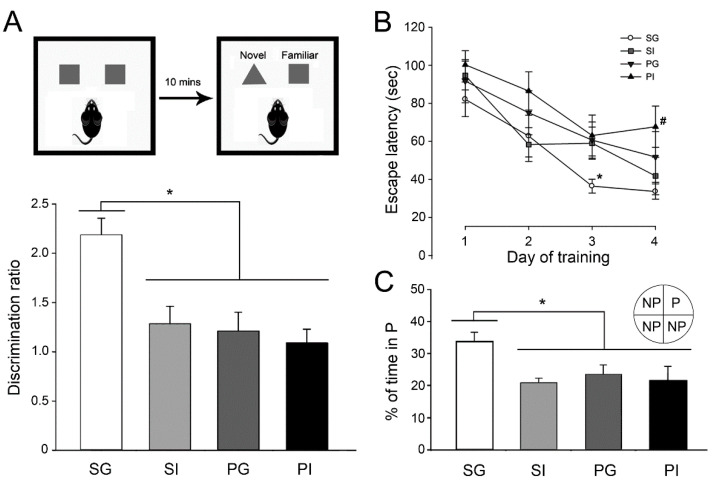
Tests of learning and memory. (**A**) The novel object recognition test paradigm was used to evaluate short-term object recognition memory. (**B**) Spatial learning and memory were evaluated using the Morris water maze test. (**C**) Spatial memory was evaluated in the probe test, in which the platform was removed. P: platform region during the training phase. NP: non-platform region. Results are mean ± SEM (*n* = 6–10 per group). * *p* < 0.05. ^#^ *p* < 0.05 compared to the SG group.

We also examined the performances of mice in learning and memory aspects. In the novel object recognition test, mice in the SI, PG and PI groups exhibited an impaired short-term object recognition memory function (Figure 4A), which is caused by either manipulation (F_(3,26)_ = 6.35, *p* < 0.05). In the Morris water maze test, mice in SI, PG and PI groups showed significant deficits in spatial learning (Figure 4B, day 3) and spatial memory (Figure 4C, probe test). At the end of the learning phase, day 4, mice in the PI group took a greater amount of time to find the platform than the mice in control SG group (Figure 4B).

### 2.2. Histological Evaluations

**Figure 5:**
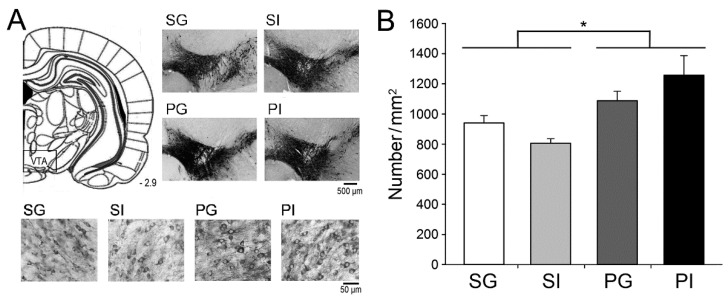
Density of dopaminergic neurons in the VTA. (**A**) Dopaminergic neurons in the VTA were revealed using tyrosine hydroxylase (TH) immunohistochemistry. (**B**) The density of TH-positive neurons in VTA was measured. Results are mean ± SEM (*n* = 5–10 mice per group). * *p <* 0.05.

The mesocorticolimbic DA system plays an important role in emotional and cognitive function [40,41,42] as well as in the stress-coping reaction [45]. We then quantified the number of DAergic neurons in the midbrain VTA using tyrosine hydroxylase (TH) immunohistochemistry (Figure 5A). Mice born to poly I:C-exposed mothers exhibited increased TH-positive cells in the VTA than SG controls (Figure 5B). This effect was independent of social isolation treatment (F_(1,25)_ = 17.86, *p* < 0.001).

**Figure 6:**
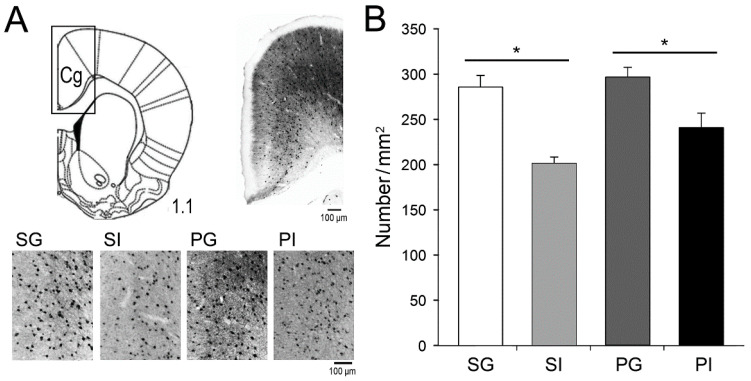
Density of parvalbumin (PV)-positive neurons in the mPFC. (**A**) PV-positive neurons in the mPFC (Cingulate cortex, Cg) were revealed using immunohistochemistry and (**B**) the density was measured. Results are mean ± SEM (*n* = 8–12 mice per group). * *p <* 0.05.

Reductions in GABAergic cortical neurons, especially parvalbumin (PV)-positive neurons, are evident in schizophrenic patients [48,49]. Therefore, we quantified the number of PV-expressing neurons in the cortex, particularly the cingulate in the medial prefrontal cortex (mPFC) (Figure 6A), which is important for numerous brain functions [50,51,52,53]. The number of PV-positive neurons in the mPFC was found to be significantly reduced in the isolation-reared groups, when compared to the group-reared group (Figure 6B). The main effect was attributed to social isolation (F_(1,34)_ = 20.33, *p* < 0.001). No significant interaction between the two manipulations was observed in this test.

**Figure 7:**
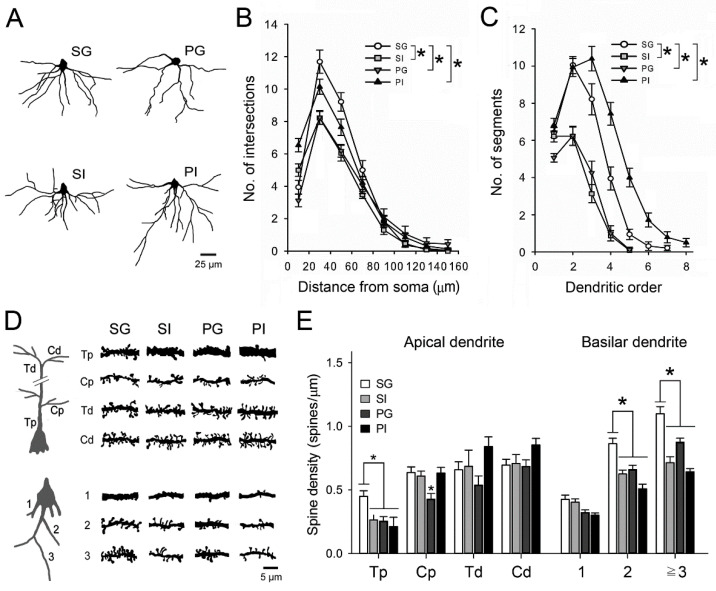
Dendritic features of layer II/III pyramidal mPFC neurons. (**A**) Somata and basilar dendrites of Golgi−Cox impregnated pyramidal neurons were reconstructed. Spines are omitted in this illustration. (**B**) The complexity of basilar dendrites was estimated using the concentric sphere method of Sholl and (**C**) the number of dendritic segments. (**D**) Dendritic segments obtained from apical and basilar dendrites are illustrated. (**E**) In the apical dendrites, fragments were collected from the trunk (T) and collaterals (C) from the proximal (<100 μm from the soma) and distal (near the surface) regions. In the basilar dendrites, fragments were classified by the dendritic order. The densities of dendrites in different dendritic segments were measured. Results are mean ± SEM. * *p* < 0.05.

**Figure 8:**
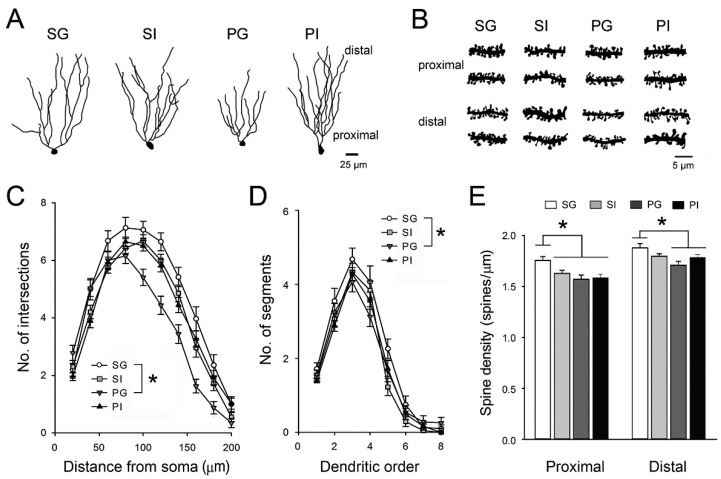
Dendritic structure of DG granule cells. (**A**) Examples of Golgi−Cox-impregnated DG granule cells were obtained from the four groups of animals. (**B**) Dendritic segments were obtained from the proximal (<50 μm from soma) and distal (>150 μm from soma) regions of DG granule cells. (**C**) The complexity of dendrites was estimated using the concentric sphere method of Sholl (**D**) and the number of dendritic segments. (**E**) Spine densities in the proximal and distal regions of granule cells were quantified. Results are mean ± SEM. * *p* < 0.05 compared to the SG group.

We further examined the morphological features of neurons in the cingulate cortex of the mPFC (Figure 7) and hippocampus (Figure 8), which play significant roles in sensorimotor gating, short-term object recognition memory, as well as spatial learning and memory. Golgi-stained layer II/III mPFC neurons were collected and reconstructed (Figure 7A). The number of intersections, bifurcation nodes and terminal ends of basilar dendrites in layers II/III pyramidal mPFC neurons were reduced in the SI and PG groups, when compared to the SG group (Table 1, Figure 7B,C). However, increased numbers of bifurcations, terminal ends and segments were observed in neurons of the PI group (Table 1, Figure 7C). The density of dendritic spines was further quantified (Figure 7D). In mice of SI, PG and PI groups, reduced spine density was noted in both proximal apical trunk and basilar dendrites, compared to the SG group (Figure 7E).

The dendritic features of granule cells in the hippocampal dentate gyrus (DG) (Figure 8A,B) were also examined. Dendritic complexity was reduced in DG neurons of all experimental groups, especially the PG group, compared with the SG group (Table 2, Figure 8A,C,D). Fewer dendritic spines were also found in DG neurons in the SI, PG and PI groups (Figure 8E). Our morphological data demonstrated that both manipulations affected the dendritic architecture of mPFC and DG neurons, which may lead to impaired synaptic transmission and integration and contribute to the decline of cognitive functions.

## 3. Discussion

Schizophrenia is a multifactorial disease. The current study examined the impact of maternal infection (first hit) combined with postweaning social isolation (second hit) as a two-hit mouse model for schizophrenia. We performed behavioral tests to assess the emotion, cognitive function and memory, immunohistochemistry of TH-positive and PV-positive neurons in the VTA and mPFC regions, respectively, and characterized the structural complexity of neurons in the mPFC and DG. Analyzing the behavioral test, neurochemical and morphological profile of neurons involved in the mesocorticolimbic dopamine system provides a clear picture of using two hits for modelling schizophrenia in mice.

### 3.1. Synergistic Effects of the Two Hits on Stress-Coping Response

A synergistic effect of prenatal infection and postnatal social isolation occurred in the forced swim test, in which the immobility time was reduced in mice of the PI group. The degree of immobility in the forced swim test would reflect the level of depression [43]. The mice in the PI group could be understood as less depressive. However, since the anxiety level of these mice was not altered, we may consider an alternative explanation. Performance in the forced swim test could reflect the coping response to a stressful situation [45] and our results suggested that the stress-coping mechanisms in PI mice were altered. Faced with life adversity or stress, one should deal with it by using suitable coping strategies. It is clear that the stress-coping strategies adopted by schizophrenia patients differ greatly from healthy subjects and ineffective coping strategies may endorse the expression of psychotic symptoms [54]. The choice and switching of coping strategies require proper cognitive flexibility; unfortunately, cognitive flexibility is diminished in patients with schizophrenia [55]. Reduced immobility in our two-hit model may replicate the cognitive inflexibility in schizophrenia patients. Both active and passive coping strategies are used in the forced swim test [45]. When a mouse was placed in the water tank, active coping behaviors including swimming, struggling or climbing were exhibited; however, when the inescapability was recognized, passive coping behaviors such as floating or immobility were performed to save energy. In this regard, reduced immobility in mice of PI group was considered as inflexibility in the stressful situation or a poor stress-coping response.

Both the mPFC and VTA are involved in the neural circuits associated with the transition between active and passive behavioral states [53,56]. Activation of the VTA-mediated mesoaccumbens DA circuit enhances the active coping behaviors whereas inhibition of dopamine release is associated with passive coping reactions [56,57]. In our model, mice in the PI group exhibited greater active coping behaviors which is closely related to the increased DA neuron density in the VTA. On the other hand, reduced mPFC PV neurons might affect the mPFC activity and the transition between active and passive coping behaviors. Although alterations in VTA DA neurons and mPFC PV neurons were caused by maternal infection and postweaning social isolation, respectively, these changes converged in mice in the PI group and, at least in part, provided a structural base for impaired stress-coping response. Interestingly, PI mice exhibited reduced locomotor activity in the open field test, which suggests that the stress-coping responses are context-dependent. Stress-coping skills in schizophrenia patients are an important issue in diagnosis and treatment [58]. Establishing an association between stress-coping mechanisms and the pathogenesis of mental illness is valuable. We may extend our explorations into different aspects of stress and the corresponding coping strategies and even the gender issues in the future.

An earlier study conducted by Deslauriers et al. examined the synergistic effects of maternal infection and postweaning isolation and increased immobility time after repeated exposures (5 consecutive days) to the forced swim test was reported as helplessness-like, suicide-trait behavior [59]. The discrepancy between this study and our current results may be attributed to the differences of experimental manipulations such as the dose and timing of poly I:C administration, the date of weaning as well as the protocol of the forced swim test. Despite the discrepancy, both studies demonstrated impaired stress-coping reactions by similar two-hit models.

### 3.2. The Mesocorticolimbic System Is Sensitive to Experimental Manipulations

The mesocorticolimbic system transmits DA from the VTA to the mPFC and limbic structures, such as the hippocampus. The dysregulation of DA function may be attributed to schizophrenia [40,41,42]. The current study demonstrated an increased number of TH-positive DA neurons in the VTA in offspring born to poly I:C-treated mothers regardless of the social experience during adolescence, which indicates a critical prenatal period for DA neuronal development in the VTA [28].

PV-expressing GABAergic neurons are fast-spiking interneurons that play a role in synchronizing the activity of excitatory neurons, which is essential for emotion and cognition, and they are also associated with various brain disorders [48,49]. Our data demonstrated a reduction of PV-positive GABAergic neurons in the mPFC of isolated mice, which may contribute to their impaired coping reactions and cognitive dysfunction. Remarkably, a reduction of the GABA synthesizing enzyme GAD67 was reported in other nongenetic two-hit models of schizophrenia [29,60]. These results support the susceptibility of the GABAergic system in mental disorders.

Dendritic features in neurons of mesocorticolimbic structures, such as the mPFC and hippocampus, are also sensitive to experimental manipulations. Altered dendritic arborization and spine density were noted in prenatal infection [28,61] and postweaning isolation models [36,62]. Each single manipulation in the present study affected the complexity of dendritic arbors of layers II/III mPFC pyramidal neurons. Notably, the number of dendritic branches increased in the two-hit condition, which may be a compensatory response to the convergent insults. However, whether the increase in dendritic branching in PI mice was associated with their altered stress-coping behavior is not clear. Reduced spine density was also evident in all three experimental groups, which is similar to the findings in postmortem brains of schizophrenic patients [63,64]. To the best of our knowledge, this report is the first morphometric study of dendritic architecture in adult offspring of this two-hit animal model of schizophrenia. These structural defects might correlate with the functional deficits.

Significant defects were noted in mice of all three experimental groups in the novel object recognition test and the probe test of water maze task. These results suggested that the consequences of two insults converge to some common brain substrates that are sensitive to either prenatal or postnatal manipulation, and a single hit is sufficient to produce severe functional impairments in these tests. Take the novel object recognition test as an example, the discrimination ratios in mice of SI and PG groups were close to 1, meaning that under the condition of either single hit, these mice are not able to differentiate the novel object from the familiar one. Combination of the two hits therefore failed to further exacerbate the deficit. A similar principle could be applied to the results in the probe test of the water maze task.

### 3.3. Superiority of This Two-Hit Non-Genetic Mouse Model of Schizophrenia

This two-hit non-genetic mouse model of schizophrenia has it unique merits. For instance, manipulations may be conducted in definite time points or during a specific time period. The critical time window of the pathogenic changes may be revealed. In the present model, the increase of VTA DA neurons resulted from prenatal manipulation (poly I:C exposure), and the reduction in PV-positive neurons in the mPFC was largely attributed to adolescent insult (postweaning social isolation). Notably, synergistic effects of the two insults on the forced swim test paradigm were observed in the current model, which may replicate the impaired stress-coping skill and cognitive inflexibility in schizophrenia patients. Establishing a connection between the underlying mechanism of the stress-coping reaction and the pathogenesis of mental illness is valuable. These results also highlight the vulnerability of the adolescent period in the pathogenesis of mental disorders [65,66]. A paradoxical change in dendritic branching was noted in the mPFC of PI mice, which indicates a critical time window of structural plasticity during adolescence. This may also provide a therapeutic time window for the treatment of neurodevelopmental illnesses [67].

In the present study, the behavioral tests and histochemical examinations were conducted right after the social isolation paradigm. Some structural and functional defects subsequent to early insults might occur later in life [68]. The long-term effects of the two hits should be further addressed.

## 4. Materials and Methods

### 4.1. Animals and Treatments

Pregnant C57BL/6J mice received either a single i.p. injection of poly I:C (2 mg/kg, Sigma-Aldrich, St. Louis, MO, USA) or vehicle (saline) on gestation day (GD) 9. To reduce the variation and issue of maternal care, we used experienced females in our study. Both saline- and poly I:C-injected mice were second- or third-time mothers. Ten litters from saline- and poly I:C-injected mothers were used. Pups were weaned at postnatal day (PD) 28. In each litter, male offspring were randomly assigned into group-rearing (3–5 mice per cage) or isolation-rearing groups. Behavioral tests and histological examinations were conducted after P56. Mice were kept in the Laboratory Animal Center of the College of Medicine, National Taiwan University, under a 12:12 light-dark cycle with free access to food and water. All animal handling was performed in accordance with a protocol approved by the Institutional Animal Care and Use Committee (IACUC) of the National Taiwan University (IACUC approval number: 20130078, 26 March 2013).

### 4.2. Behavioral Tests

Behavioral experiments were conducted during the light phase (between 9 am and 4 pm). One cohort of mice were tested in the open field and then used for novel object recognition test, the interval was 2 days. Two days after the novel object recognition test, these mice were further used for histochemical examinations. Another cohort of mice were used for the three-chamber test, prepulse inhibition test and forced swim test. Still another cohort of mice were used in elevated plus maze test and Morris water maze. The order of behavioral tests was fixed and the interval was 2–3 days. Before the tests, mice were brought to the testing room for habituation (>30 min). Animal behaviors were recorded on a video camera and analyzed using Topscan LITE software (Clever system, Reston, VA, USA).

#### 4.2.1. Three-Chamber Test

The sociability of mice was evaluated in this test. A Plexiglas cage (40 × 60 × 22 cm) was divided into three equal regions. Before the test, a mouse was allowed to habituate in the chambers for 10 min. For the sociability test, we confined the test mouse in the central chamber and place a target mouse in a porous pen holder in one side chamber. An empty porous pen holder was placed in another side chamber. After the removal of the doorways between the central and side chambers, the time spent in each chamber by the test mouse was recorded for 5 min and analyzed.

#### 4.2.2. Open Field Test

This test was used to evaluate general locomotor activity levels and anxiety in rodents. After habituation, an individual mouse was placed in the open field (40 cm × 40 cm) for 10 min. The middle part (24 cm × 24 cm) of the open field was defined as the central region, and the remaining areas were the peripheral region. The distance travelled and time spent in the central and peripheral regions were analyzed.

#### 4.2.3. Elevated Plus Maze Test

This test was used to evaluate anxiety-related behaviors, which are associated with the symptoms of schizophrenia, as previously described [57]. Briefly, a custom-made maze consisting of two open arms (40 cm × 6 cm) crossed at a right angle by two closed arms located 50 cm above the floor. An individual mouse was placed on the central platform and allowed to move freely for 10 min. The distance travelled and time spent in the open arms, closed arms and central platform were quantified.

#### 4.2.4. Forced Swim Test

It is a common test to assess the depression-like symptom of schizophrenia based on stress-coping behavior [53]. This test was conducted as previously described [57]. In brief, an individual mouse was confined in a Plexiglas cylinder filled with water (25 °C) for 8 min. Three behavioral parameters, including struggling, immobility and swimming, were characterized during the last 6 min of the test period.

#### 4.2.5. Prepulse Inhibition Test

The sensorimotor gating function of mice was assessed using the prepulse inhibition (PPI) of the acoustic startle reflex. Impairment of PPI has been noted in patients and various animal models of schizophrenia [46,47]. The PPI test was conducted using SR-Lab system (San Diego Instruments, San Diego, CA, USA) as previously described (Ko et al., 2014). In brief, an individual mouse was acclimated to the apparatus for 5 min followed by a test of 64 trials including pulse (120 dB)-alone trials, prepulse (68, 71 or 77 dB)-plus-pulse trials, and no-stimulus trials. The percentage of PPI was calculated as: PPIn (%) = 100 × ((pulse-alone) − (prepulse-plus-pulse))/pulse-alone, where n is the magnitude (dB) of the prepulse.

#### 4.2.6. Novel Object Recognition Test

Impaired short-term object recognition memory is associated with the cognitive symptom of schizophrenia. This function was evaluated in mice using the novel object recognition test as previously described [28]. In brief, a mouse was placed in an open field twice daily for 2 days during the habituation phase. The mouse was then placed in the open field and presented with a pair of identical objects, allowed to freely explore for 8 min. After exploration, the mouse was returned to the home cage and kept for 10 min. Subsequently, the mouse was returned to the open field in which one of the original objects was replaced by a novel one. The mouse was allowed to freely explore again for 8 min. The time spent on exploration behaviors was quantified. The discrimination ratio ((novel object exploration time)/(familiar object exploration time)) was calculated.

#### 4.2.7. Morris Water Maze

Deficits in spatial learning and memory are noted cognitive syndromes in schizophrenia patients which can be evaluated in rodents using the Morris water maze test. Briefly, a pool (100 cm in diameter) was filled with milky water, and a platform was placed at the center of a fixed quadrant, 2 cm below the water surface. A mouse was released into the pool with its head facing the wall and allowed to search for the hidden platform using three visual cues. Each mouse was allowed to stay on the platform for 30 s. Four trials of different starting points were administered per day for 4 consecutive days (learning phase). The latency and swimming path from the release to the platform was recorded and measured for all trials. The spatial memory was assessed in a probe trial one day after the last training trial. The hidden platform was removed in the probe trial, and the swimming path of each mouse was recorded and measured.

### 4.3. Histology

Mice were perfused with phosphate-buffered saline (PBS) followed by the fixative (4% paraformaldehyde in phosphate buffer, pH 7.4). Brains were postfixed in the same fixative overnight and maintained in PBS containing sodium azide (0.1%). To avoid the influence of behavioral tests on the histochemical and morphological features of mice, samples for Golgi stain were collected from those naïve to behavioral test, while sections for immunochemical examinations were obtained from mice used in open field test and novel object recognition test.

#### 4.3.1. Immunohistochemistry

Coronal sections of the brain were cut at a thickness of 30 µm using a vibrating microtome (vibratome 1000, the Vibratome Company, St. Louis, MO, USA). Primary antibodies, including mouse anti-tyrosine hydroxylase (TH; 1:2000; Sigma-Aldrich) or mouse anti-Parvalbumin (PV) (1:2000; Sigma-Aldrich), were used. Biotinylated goat anti-mouse IgG (1:500 the Jackson ImmunoResearch Laboratories, West Grove, PA, USA) was used as the secondary antibody. Immunoreactivity was amplified using an avidin-biotin complex system (1:100, Vector Labs, Burlingame, CA, USA). TH is the key enzyme for dopamine synthesis, and TH immuno-histochemistry was used to label dopaminergic neurons. PV immunostaining was used to label PV-positive GABAergic interneurons. The densities of immunopositive cells were estimated using StereoInvestigator system (MicroBrightField Bioscience, Williston, VT, USA) [35].

#### 4.3.2. Golgi−Cox Impregnation and Morphometric Analyses

Brain samples were immersed in the impregnation solution of the FD Rapid Golgi Stain kit (NeuroTechnologies, Ellicott City, MD, USA) and cut at a thickness of 150 μm using a vibratome. Stacks of images of Golgi−Cox-impregnated layer II/III pyramidal neurons in the medial prefrontal cortex (mPFC) and granule cells in the dentate gyrus (DG) were acquired with the StereoInvestigator system. The morphology of selected neurons was reconstructed and analyzed using Neurolucida software (Microbrightfield Bioscience) [28]. The density of the dendritic spines was determined and expressed as the number of spines per μm of the dendritic length.

### 4.4. Statistical Analyses

Behavioral tests and immunohistochemistry data were analyzed using two-way analysis of variance (ANOVA) to evaluate the interaction of prenatal infection and social isolation, followed by Fisher’s least significant difference (LSD) post hoc comparisons. Repeated measures ANOVA was used for the learning phase in the Morris water maze. Morphological analysis data were analyzed using one-way ANOVA, followed by LSD post hoc comparisons. Data are expressed as the mean ± standard error of the mean (SEM). Statistical significance for all comparisons was set at *p* < 0.05.

## 5. Conclusions

Our results demonstrated that an early prenatal risk factor interacts with later adolescent adversity and render a defect in stress-coping reaction in mice. Our findings also indicate adolescence as a critical pathogenic or therapeutic time window for mental illness, such as schizophrenia.

## Figures and Tables

**Table 1:** Morphometric features of basilar dendrites in layer II/III pyramidal neurons in the mPFC. SG: *n* = 19 neurons, SI: *n* = 23 neurons, PG: *n* = 16 neurons, PI: *n* = 32 neurons; neurons were obtained from 6 mice in all groups. Data are presented as the mean ± SEM. * *p* < 0.05 compared to the SG group.

	SG	SI	PG	PI
**Primary dendrites**	6.42 ± 0.27	6.22 ± 0.30	5.06 ± 0.23 *	6.82 ± 0.34
**Bifurcation nodes**	11.79 ± 0.80	5.13 ± 0.39 *	5.81 ± 0.56 *	15.36 ± 1.11 *
**Terminal ends**	18.32 ± 0.78	11.39 ± 0.44 *	10.88 ± 0.59 *	24.04 ± 1.41 *
**Total dendritic length (m)**	768.1 ± 34.35	552.6 ± 23.85 *	583.8 ± 47.84 *	754.2 ± 30.92

**Table 2:** Morphometric features of DG granule cells. Data are presented as the mean ± SEM. SG: *n* = 31 neurons, SI: *n* = 49 neurons, PG: *n* = 32 neurons, PI: *n* = 65 neurons; neurons were obtained from 6 mice in all groups. * *p *< 0.05 compared to the SG group.

	SG	SI	PG	PI
**Bifurcation nodes**	7.65 ± 0.45	6.27 ± 0.23 *	6.44 ± 0.31 *	6.46 ± 0.22 *
**Terminal ends**	9.77 ± 0.64	8.01 ± 0.26 *	8.28 ± 0.36 *	8.02 ± 0.19 *
**Highest order**	5.16 ± 0.23	4.53 ± 0.11 *	4.88 ± 0.23	4.72 ± 0.13
**Total dendritic length (m)**	1094.79 ± 50.42	954.3 ± 26.53 *	835.4 ± 34.14 *	956.7 ± 25.78 *

## Abbreviations

ANOVA	analysis of variance
DA	dopamine
DG	dentate gyrus
GABA	γ-Aminobutyric acid
GD	gestation day
LSD	least significant difference
mPFC	medial prefrontal cortex
PD	postnatal day
PG	poly I:C-group
PI	poly I:C-isolation
poly I:C	poly-inosinic/cytidylic acid
PPI	prepulse inhibition
PV	parvalbumin
SEM	standard error of the mean
SG	saline-group
SI	saline-isolation
TH	tyrosine hydroxylase
VTA	ventral tegmental area
